# A Subtle Sighting: Acute Midbrain Infarction Presenting as Isolated Medial Rectus Palsy

**DOI:** 10.7759/cureus.88249

**Published:** 2025-07-18

**Authors:** Salomon Chamay, Ermias Greffie, Francisco J Gallegos Koyner, Nelson Barrera, Scott Segan

**Affiliations:** 1 Internal Medicine, SBH Health System, New York City, USA; 2 Internal Medicine, St. Barnabas Hospital Health System, New York City, USA; 3 Neurology, SBH Health System, New York City, USA

**Keywords:** binocular diplopia, isolated oculomotor palsy, midbrain infarction, oculomotor nerve (cn iii) palsy, stroke

## Abstract

Isolated ocular motor palsy involving one extraocular muscle is a rare clinical manifestation of cerebrovascular accident and is more commonly attributed to orbital trauma and muscular disease. Oftentimes, diplopia is the only symptom reported by patients. Differentiating between monocular and binocular diplopia is a fundamental step in the diagnostic pathway. Furthermore, a cautious and detailed neurological examination is required. We present a case of a 64-year-old male with isolated left medial rectus palsy secondary to an acute midbrain infarction, with no other manifestations or symptoms of acute stroke.

## Introduction

Acute ischemic stroke is defined as a sudden neurological dysfunction resulting from focal brain ischemia lasting greater than 24 hours or evidenced by acute infarction on brain imaging [1]. In the United States, stroke is the fifth leading cause of death and accounts for an estimated annual cost exceeding $70 billion [1]. Isolated extraocular palsy is most commonly caused by muscular disease or orbital lesions, but rarely can also present as a manifestation of acute stroke [2]. Diplopia is often the initial presenting symptom, and a careful neurologic examination is warranted in order to ascertain the diagnosis [2]. Differentiating between binocular and monocular diplopia is crucial and allows for the stratification of patients into two groups: ocular misalignment or local eye disease and refractive pathology, respectively. Ocular misalignment warrants attentive assessment of individual extraocular muscles and consideration of cerebrovascular pathology. We present a case of a 64-year-old male with isolated left medial rectus palsy secondary to acute midbrain infarction presenting as diplopia without other neurological signs or symptoms.

## Case presentation

**Figure 1:**
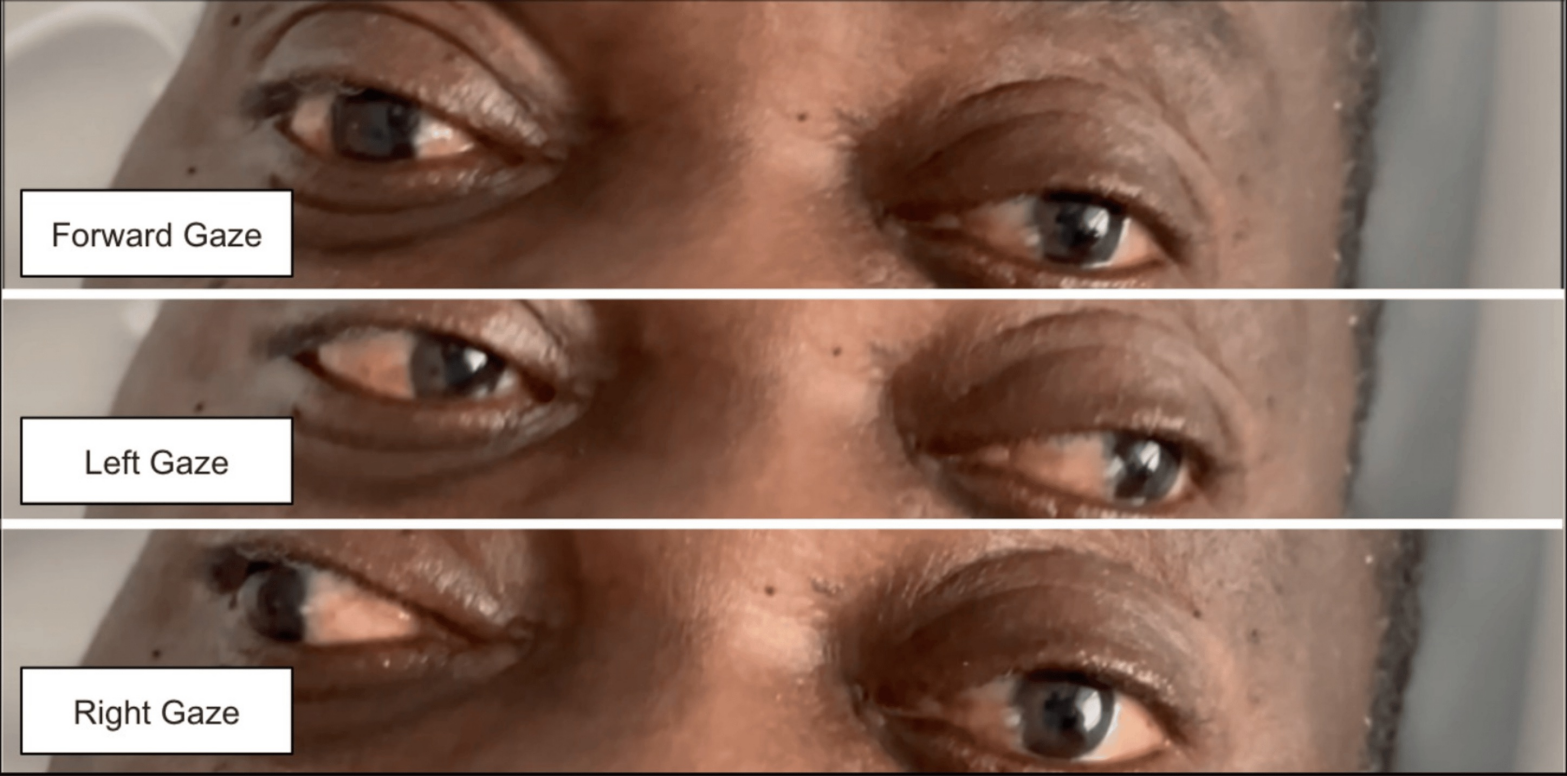
Left eye adduction deficit noted with rightward gaze (most inferior image)

A right-handed 64-year-old male with a past medical history of hypertension, chronic kidney disease (CKD) stage 3A, and previous cerebrovascular accidents without residual deficits presented to the emergency room complaining of double vision that began the night prior. He reported non-compliance with blood pressure medications. Review of systems was positive for double vision and headache. Vital signs on admission were remarkable for an elevated blood pressure of 239/129 mmHg. On neurological examination, a left eye adduction deficit was noted on rightward gaze (Figure 1). The adduction deficit persisted when examined with a closed right eye. The pupils were equal and reactive to light, ptosis was absent, and the rest of his eye movements were normal. The patient reported improvement of the diplopia on closing one eye. All other cranial nerves were intact. The patient was able to follow commands, speak fluently, and name objects. Motor strength was 5/5 throughout, and sensation was intact to light touch, temperature, and pin prick. Deep tendon reflexes were normal, and the Babinski sign was absent. A stroke alert was called, given the patient's presentation and medical history; however, thrombolytic therapy was deferred due to the patient presenting outside of the therapeutic window. Aspirin and a high-intensity statin were started.

The patient was admitted to the telemetry unit. Laboratory studies were remarkable only for an elevated creatinine of 1.3mg/dL (the patient’s baseline was 1.5-1.6 mg/dl). Hemoglobin A1c was 6.0% and low-density lipoprotein (LDL) cholesterol was 132. Troponins were negative. The electrocardiogram showed a normal sinus rhythm with voltage criteria for left ventricular hypertrophy. Echocardiogram showed left ventricular hypertrophy without other abnormalities. No significant events were recorded during telemetry monitoring.

**Figure 2:**
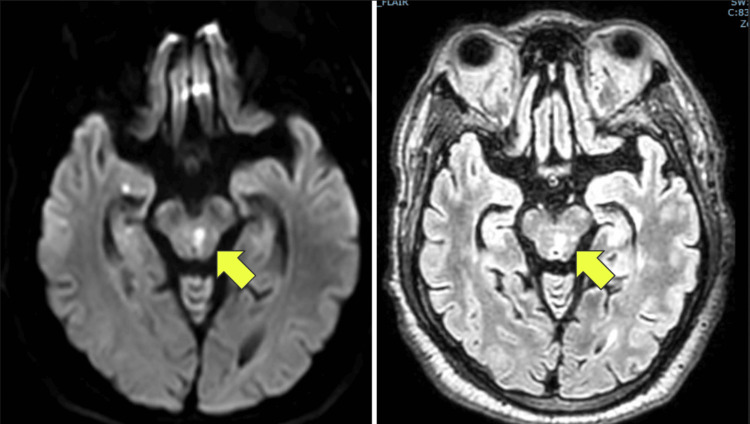
MRI brain demonstrating a lacunar infarction in the left dorsal superior pons extending up into the midbrain (yellow arrows)

Computed tomography (CT) brain was obtained and showed a lacunar infarct in the right posterior-paramedian pons and right posterior para-ventricular corona radiata of indeterminate age. MRI brain was subsequently obtained and showed diffusion restriction in the left dorsal superior pons extending up into the midbrain, demonstrating an acute small vessel infarction (Figure 2). Additionally, subacute to chronic lacunar infarcts were noted within different cerebral, brainstem, and cerebellar territories. Magnetic resonance angiogram (MRA) was negative for carotid and intracranial vessel disease. MRI of orbits was unremarkable for an anatomic abnormality of the extraocular muscles or aneurysm.

Ophthalmology evaluation revealed no intraocular abnormalities. The final diagnosis was binocular diplopia secondary to isolated left medial rectus palsy due to an acute pontine and midbrain lacunar stroke. The patient was started on aspirin, Plavix, and a high-intensity statin, and his hypertension was managed. The patient’s diplopia improved slightly throughout his hospital course, and he was subsequently discharged home. One month after hospital discharge, the patient was seen at the ophthalmology clinic and noted to have improvement of the adduction deficit. Additionally, the diplopia had resolved, and no nystagmus, pupil abnormality, or ptosis was noted at that time.

## Discussion

Diplopia or double vision is a symptom with many potential etiologies, including both ophthalmological and neurological [3]. In the United States, it is estimated that annually, approximately 805,000 ambulatory and 50,000 emergency room visits involve a chief complaint of diplopia [4]. Diplopia can be classified as monocular or binocular, and the distinction between the two has important clinical and prognostic implications. Binocular diplopia occurs when both eyes are open and disappears when one eye is covered, while monocular diplopia persists when the unaffected eye is covered [2,3]. Binocular diplopia usually indicates a problem with conjugate gaze mechanics, which could have neurologic or mechanical etiologies. In contrast, monocular diplopia occurs due to ophthalmologic causes such as dry eyes, corneal scarring, cataracts, retinal membranes, or a non-organic cause [2]. 

Acute-onset binocular diplopia can result from cerebrovascular accidents. Depending on the location, the oculomotor, trochlear, or abducens nerves can be affected, leading to abnormalities in extraocular movement. In a prospective study conducted in the United Kingdom by Rowe et al. involving a cohort of 890 patients diagnosed with stroke, ocular motor cranial nerve palsy was reported in 89 patients (10%) [5]. Of those 89 patients, unilateral third nerve palsy was reported in 23 (26%), unilateral fourth nerve palsy in 14 (16%), and unilateral sixth nerve palsy in 52 (58%) patients [5]. Patients with isolated oculomotor cranial nerve palsies attributed to stroke frequently have known atherosclerotic risk factors such as older age, hypertension, hyperlipidemia, diabetes mellitus, and tobacco use [6]. 

Small vessel pontine and midbrain infarctions can affect the oculomotor nucleus alone or together with the medial longitudinal fasciculus (MLF). The expected neurologic deficit from the involvement of the oculomotor nucleus is the classic “down and out eyes” with dilated pupils and ptosis. Damage to the MLF presents as internuclear ophthalmoplegia (INO). Isolated medial rectus palsy as a result of midbrain infarction remains an extremely rare entity. In a study of 2447 patients who sustained cerebral infarction or hemorrhage, only 0.25% presented with an isolated fascicular stroke [7]. Isolated mesencephalic ischemic lesions are hypothesized to be rare due to the rich vascular supply provided by the perforating paramedian artery [7]. However, due to its larger nucleus, the third cranial nerve is more prone to ischemic injury, and affected patients have a relatively high risk for recurrent stroke in the following year [7]. A meta-analysis conducted by Shew et al. found that ocular cranial nerve palsies carried a sixfold risk of subsequent stroke, with the highest risk occurring within the first year of the palsy [8]. Furthermore, the authors found that the highest risk was seen in CN III palsies with up to an eightfold increased risk of future stroke in this patient group [8]. Despite initial discomfort and deficit noted with oculomotor cranial nerve palsies, recovery is promising. Park et al. found that partial recovery occurred in 85.2% of patients, complete recovery of angle deviation occurred in 67.6% of patients, and the average time from onset to complete recovery of deficit was 3.5 months [9].

The oculomotor nuclear complex is composed of multiple subnuclei at the level of the superior colliculi in the ventral periaqueductal midbrain at the level of the superior colliculus [10]. Our patient’s presentation with isolated medial rectus palsy was likely due to the small infarction involving specifically the ventral portion of the oculomotor nuclear complex, which supplies the ipsilateral medial rectus muscle.

The differential diagnosis for medial rectus palsy includes INO. The proximity of the MLF to the oculomotor nucleus may result in gaze palsy, which could be a combination of INO and oculomotor palsy. However, the absence of nystagmus in the abducting eye, the inability for convergence, and the persistence of gaze palsy after covering the unaffected eye in our patient’s case support isolated medial rectus palsy rather than INO. Al-Sofiani et al. described a similar case of midbrain infarction resulting in an isolated medial rectus palsy with a similar argument as to why the diagnosis was favored over INO [11]. The absence of ptosis and other gaze palsy makes neuromuscular junction disorders like myasthenia gravis unlikely. A normal MRI exam of the orbits also ruled out any other intra-orbital mechanical etiologies.

Case reports describing isolated medial rectus palsy secondary to stroke are rare. In general, pupillary sparing oculomotor palsy without other associated neurologic abnormalities has been ascribed to microvascular ischemic neuropathy. However, different studies have shown that patients with isolated CN palsies, which are thought to be due to microvascular causes, may actually have CNS lesions, including brainstem stroke [3,5]. This could probably be due to failure to image the brain in these patients, and the number may actually be higher, as the diagnosis can easily be missed. We presented this case to highlight the importance of conducting a detailed and cautious neurological examination of the oculomotor cranial nerves. Timely identification of a deficit may prompt immediate intervention and improve outcomes in these patients.

## Conclusions

In patients with several cardiovascular risk factors, new-onset diplopia may be the initial manifestation of a cerebrovascular accident and warrants immediate brain imaging. A thorough neurological examination is paramount, including assessment of individual extraocular muscles, which may further aid with the diagnosis. Isolated extraocular palsy is a rare and subtle manifestation of a cerebrovascular accident that requires a detailed neurological examination, and if detected early, may allow for immediate intervention.
